# Supplementary material for: Dissecting features of epigenetic variants underlying cardiometabolic risk using full-resolution epigenome profiling in regulatory elements
Source: Nat Commun. 2019 Mar 14;10:1209. doi: 10.1038/s41467-019-09184-z (PMC6418220; doi:10.1038/s41467-019-09184-z)
Supplement: Supplementary file 1 — Supplementary Information [file 41467_2019_9184_MOESM1_ESM.pdf]

Dissecting features of epigenetic variants underlying cardiometabolic risk using full-resolution epigenome profiling in regulatory elements

Allum et al.

Table of Contents

Supplementary Figures **3**
Supplementary Figure 1 3
Supplementary Figure 2 4
Supplementary Figure 3 5
Supplementary Figure 4 6
Supplementary Figure 6 8
Supplementary Figure 7 9
Supplementary Figure 8 10
Supplementary Figure 9 11
Supplementary Figure 10 12
Supplementary Figure 11 13
Supplementary Figure 12 14
Supplementary Figure 13 15

Supplementary Tables **16**
Supplementary Table 1 16
Supplementary Table 2 17
Supplementary Table 3 18
Supplementary Table 4 19
Supplementary Table 5 20
Supplementary Table 6 21

Supplementary Notes **22**
Supplementary Note 1 22

Supplementary Figures

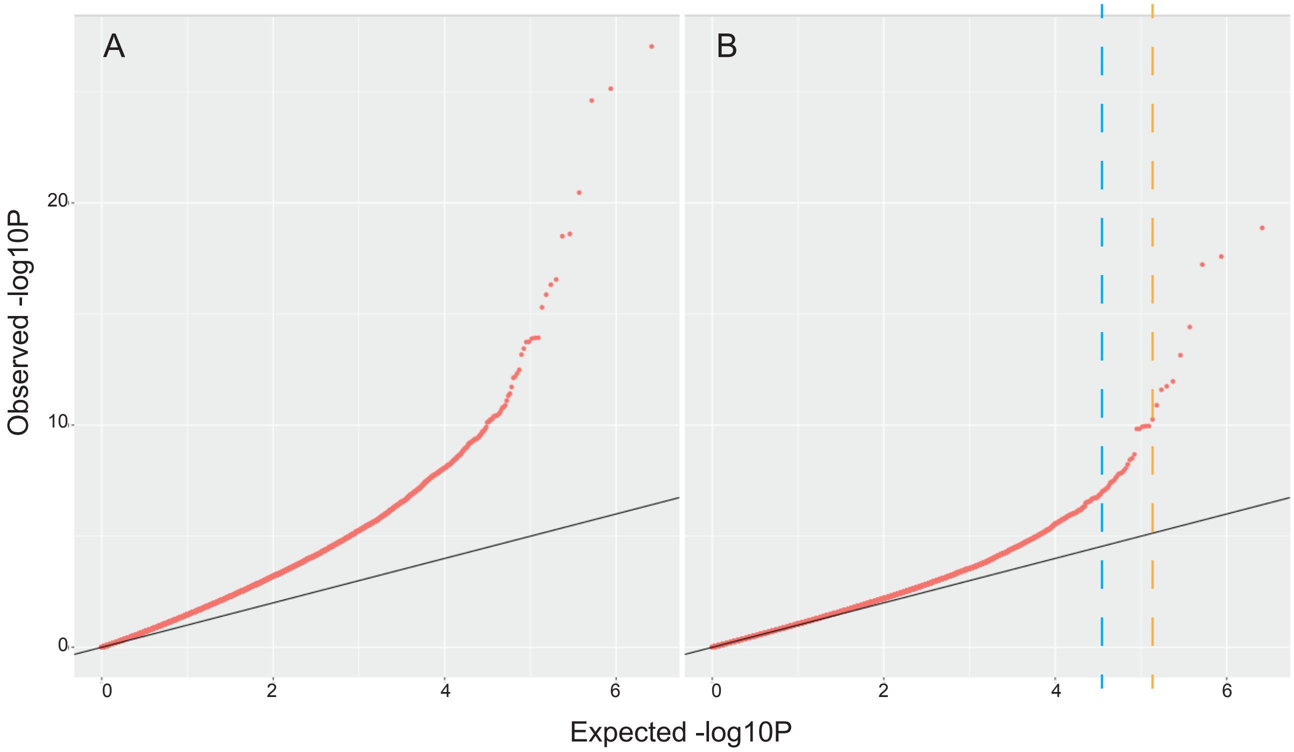

Supplementary Figure 1. QQplots for EWAS of TG to methylation associations before and after correction. Associations between triglycerides (TG) levels and methylation in visceral adipose tissue were assessed at 1,299,825 CpGs. The Bayesian method *BACON* was applied to control for bias and inflation of our test-statistics. QQplots of p-values (a) before (lambda=1.6148) and (b) after (lambda=1.0387) statistical correction are shown. FDR 10% (blue dotted line) and FDR 5% (orange dotted line) cutoffs are depicted.

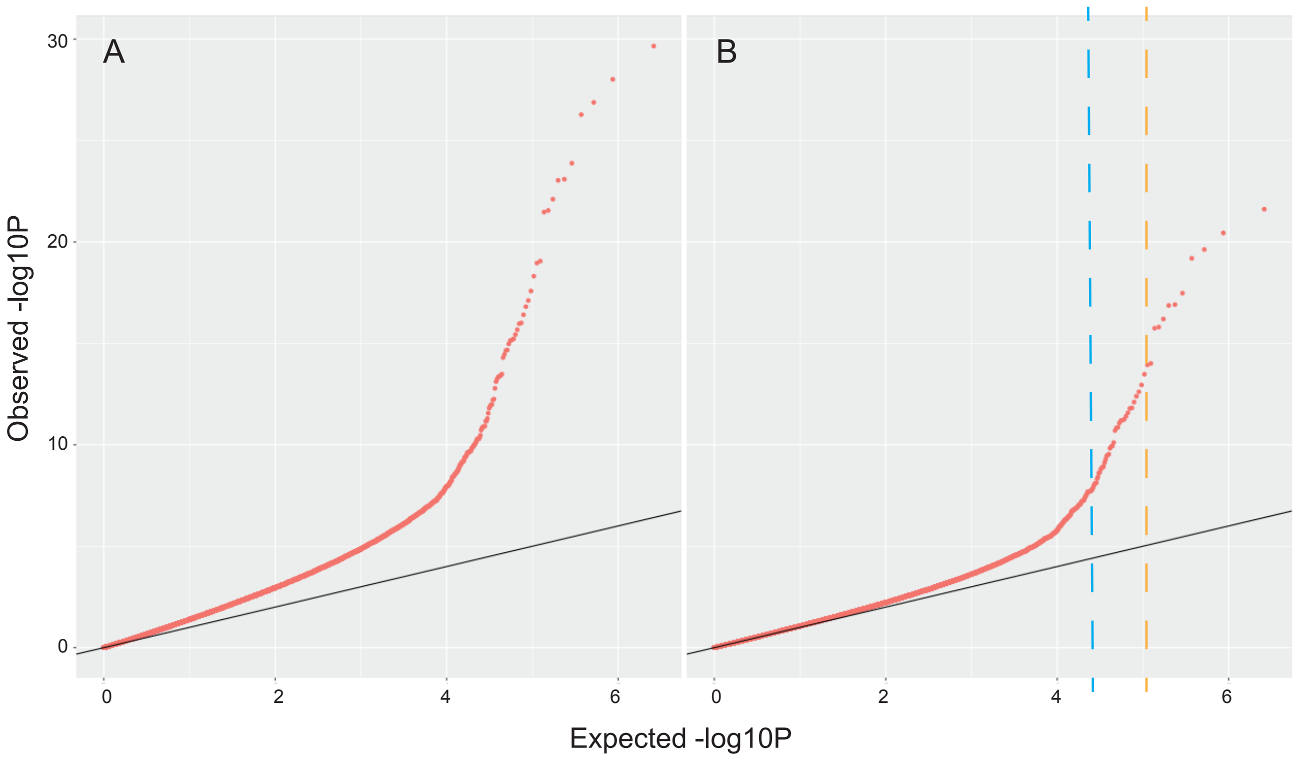

Supplementary Figure 2. QQplots for EWAS of HDL to methylation associations before and after correction. Associations between HDL-C (HDL) levels and methylation in visceral adipose tissue were assessed at 1,299,825 CpGs. The Bayesian method *BACON* was applied to control for bias and inflation of our test-statistics. QQplots of p-values (a) before (lambda=1.5232) and (b) after (lambda=1.0718) statistical correction are shown. FDR 10% (blue dotted line) and FDR 5% (orange dotted line) cutoffs are depicted.

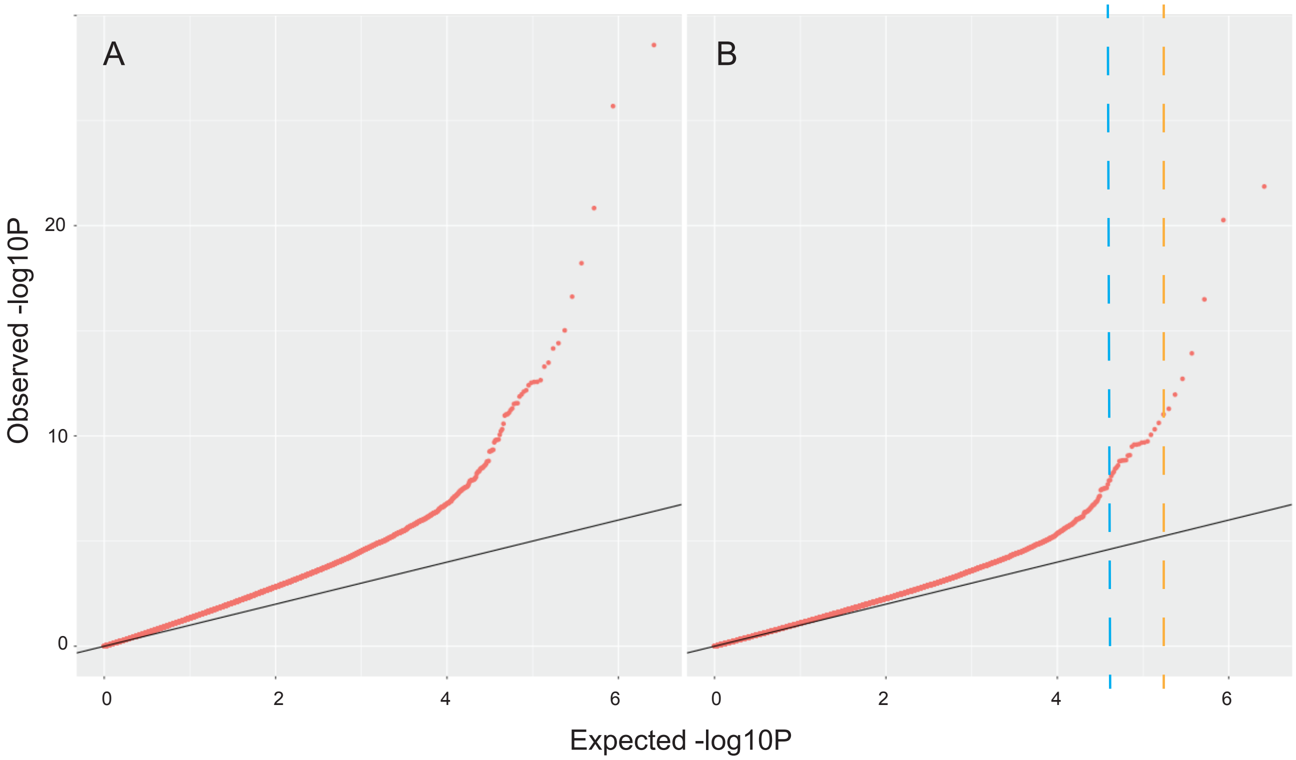

Supplementary Figure 3. QQplots for EWAS of LDL to methylation associations before and after correction. Associations between LDL-C (LDL) levels and methylation in visceral adipose tissue were assessed at 1,299,825 CpGs. The Bayesian method *BACON* was applied to control for bias and inflation of our test-statistics. QQplots of p-values (a) before (lambda=1.4465) and (b) after (lambda=1.1088) statistical correction are shown. FDR 10% (blue dotted line) and FDR 5% (orange dotted line) cutoffs are depicted.

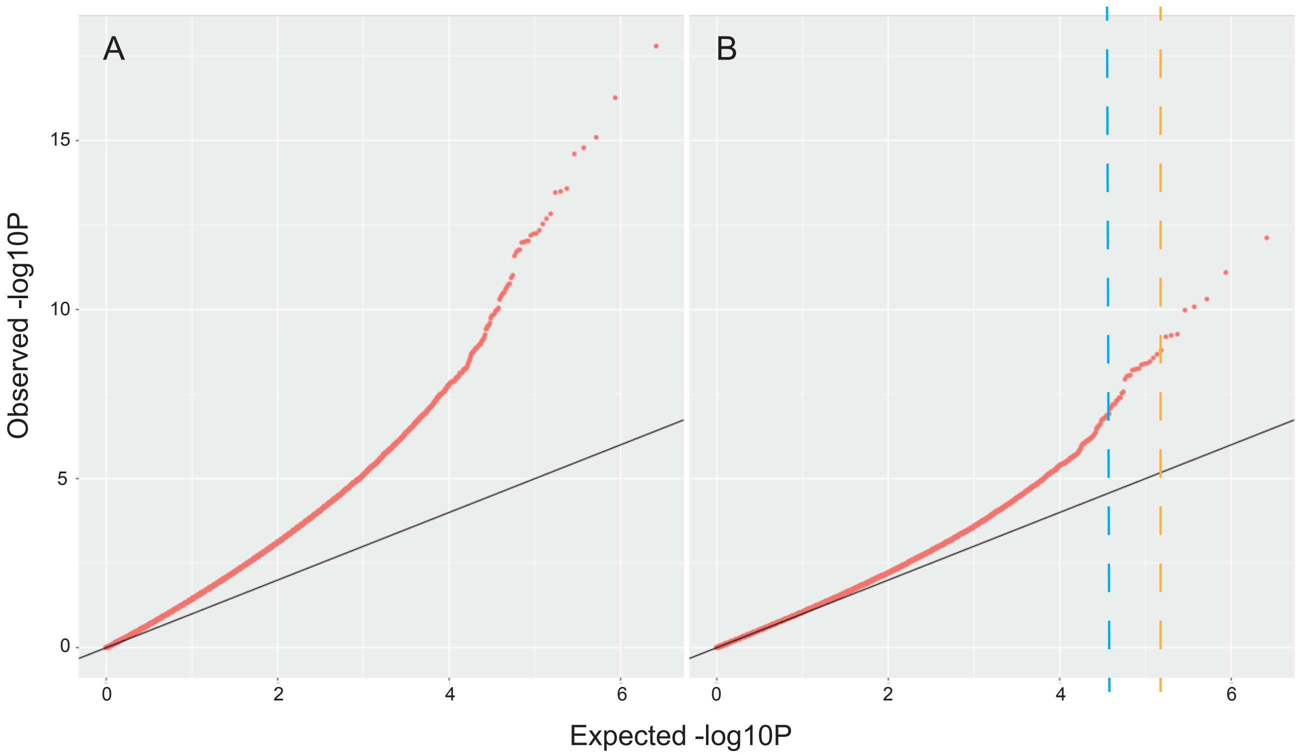

Supplementary Figure 4. QQplots for EWAS of TC to methylation associations before and after correction. Associations between total cholesterol (TC) levels and methylation in visceral adipose tissue were assessed at 1,299,825 CpGs. The Bayesian method *BACON* was applied to control for bias and inflation of our test-statistics. QQplots of p-values (a) before (lambda=1.5779) and (b) after (lambda=1.0499) statistical correction are shown. FDR 10% (blue dotted line) and FDR 5% (orange dotted line) cutoffs are depicted.

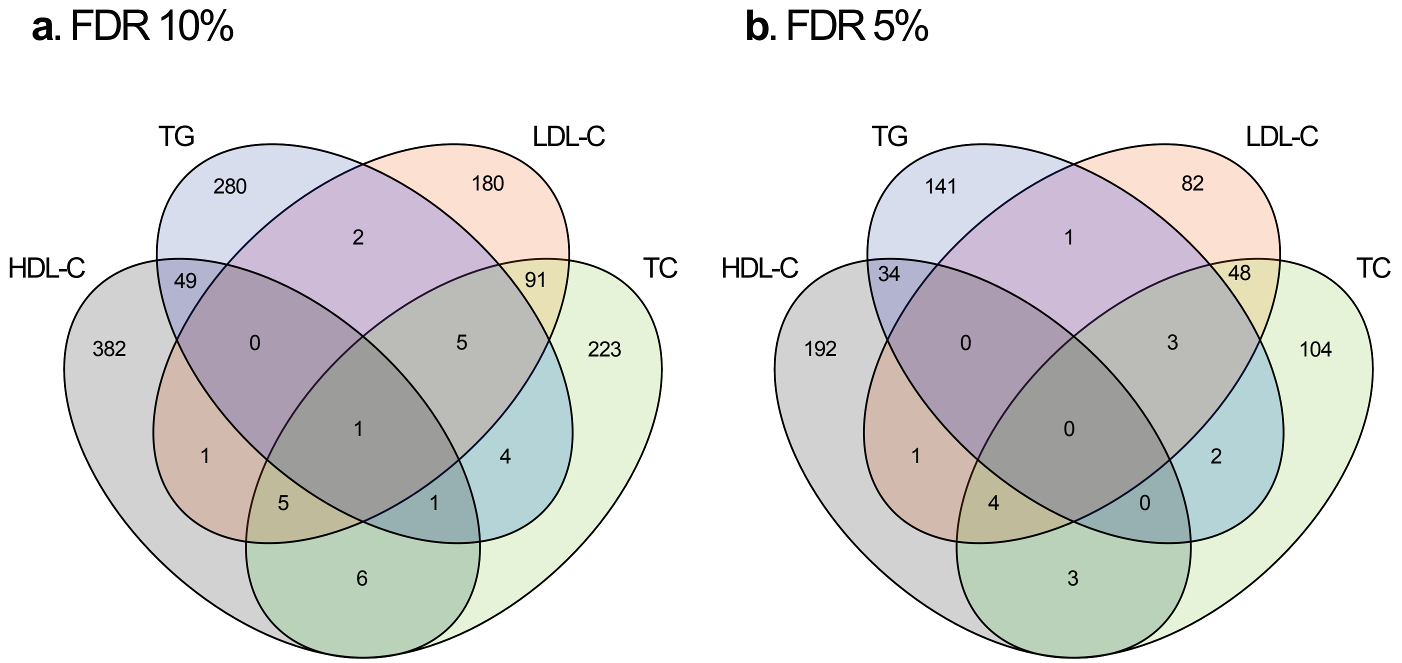

Supplementary Figure 5. Significant associations between methylation and lipid phenotypes in the discovery cohort. Associations between lipid traits (e.g. triglycerides (TG), HDL-C, LDL-C and total cholesterol (TC) levels) and CpG methylation were assessed at 1,299,825 CpGs. Overlaps between the different lipid-CpG sets are depicted in Venn diagrams for lipid-CpGs significant at (a) FDR 10% (N=1,230 lipid-CpGs) and (b) FDR 5% (N=615 lipid-CpGs).

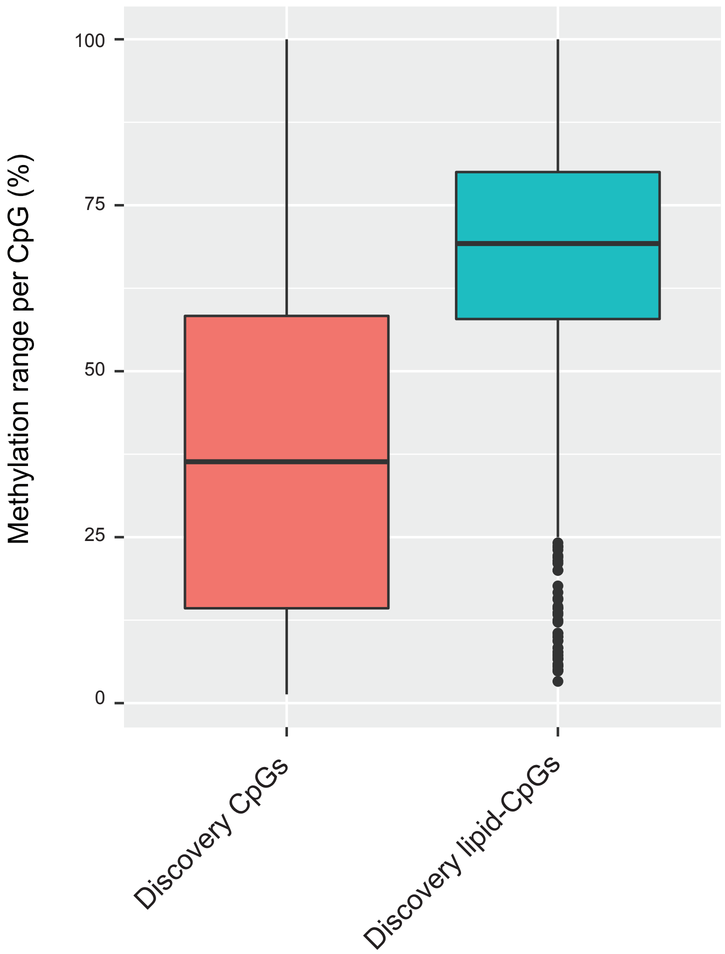

Supplementary Figure 6. Methylation range variance across CpGs within the discovery cohort. The range of methylation captured at each CpG across individuals was assessed in the discovery adipose tissue cohort. Boxplot representations of the methylation range per CpG (y-axis) are depicted for (red) all CpGs captured in the discovery cohort via the MCC-Seq method (N=1,299,825 CpGs) and (teal) lipid-CpGs significant at FDR 10% in the discovery cohort (N=1,230 CpGs).

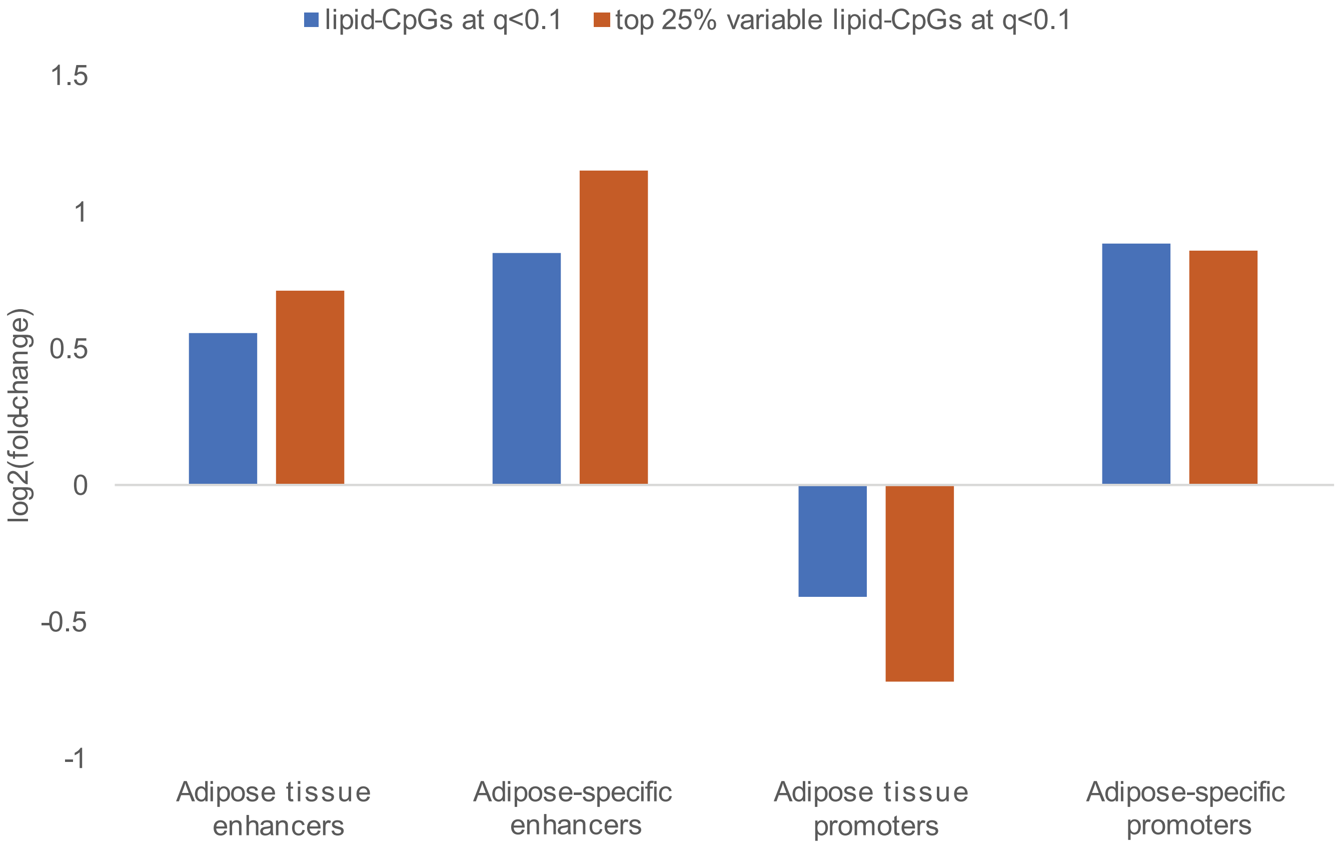

Supplementary Figure 7. Annotation of lipid-CpGs among adipose tissue regulatory elements. Discovery cohort CpGs showing association to lipid traits were mapped and annotated to adipose tissue regulatory regions. Trends observed for all lipid-CpGs (blue) and those within the top 25th percentile of methylation variability (orange) are contrasted. Significant enrichment (y-axis) of lipid-CpGs within adipose tissue putative enhancer regions (low-methylated regions; LMRs) was observed for both sets of lipid-CpGs (blue $p=6.6\text{x}10^{-13}$; orange $p=2.7\text{x}10^{-16}$), which was strengthened when limiting to adipose-unique LMRs (blue $p=9.9\text{x}10^{-13}$; orange $p<2.2\text{x}10^{-16}$). Significant depletion (y-axis) of lipid-CpGs was noted within adipose tissue putative promoter regions (unmethylated regions; UMRs; blue $p<2.2\text{x}10^{-16}$; orange $p<2.2\text{x}10^{-16}$). In contrast, enrichment was again found when restricting to adipose-unique UMRs (blue $p=8.1\text{x}10^{-11}$; orange $p=1.1\text{x}10^{-7}$). Association between lipid traits and CpG methylation was tested at 1,299,825 CpGs. Fold-change significance was calculated using Fisher's exact test.

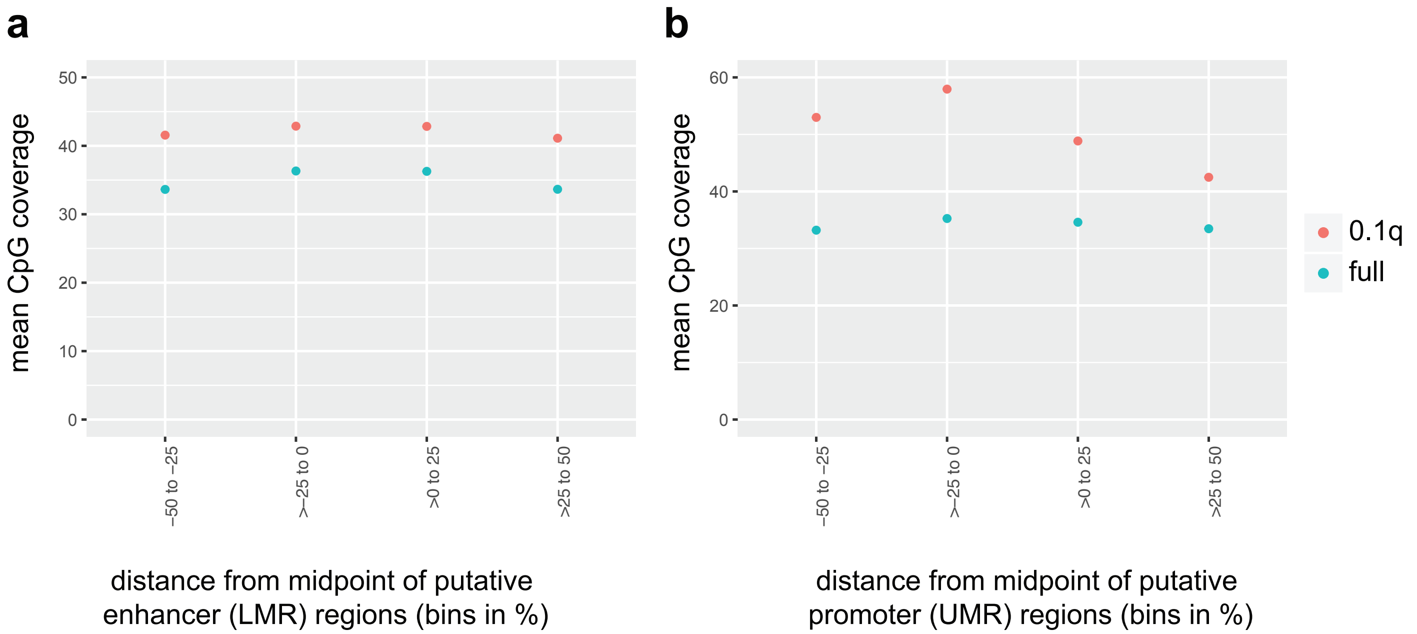

Supplementary Figure 8. Mean CpG coverage across adipose tissue regulatory elements. To account for possible biases attributable to coverage, the mean CpG coverage within 25% bins of the total distance across the elements were tabulated and plotted for (a) all CpGs (N=225,771) and lipid-CpGs at FDR 10% (N=314) mapping to LMRs and, (b) all CpGs (N=418,246) and lipid-CpGs at FDR 10% (N=225) mapping to UMRs within +/-1.5kb of transcription start sites (TSS) not depicting bivalent gene transcription orientations.

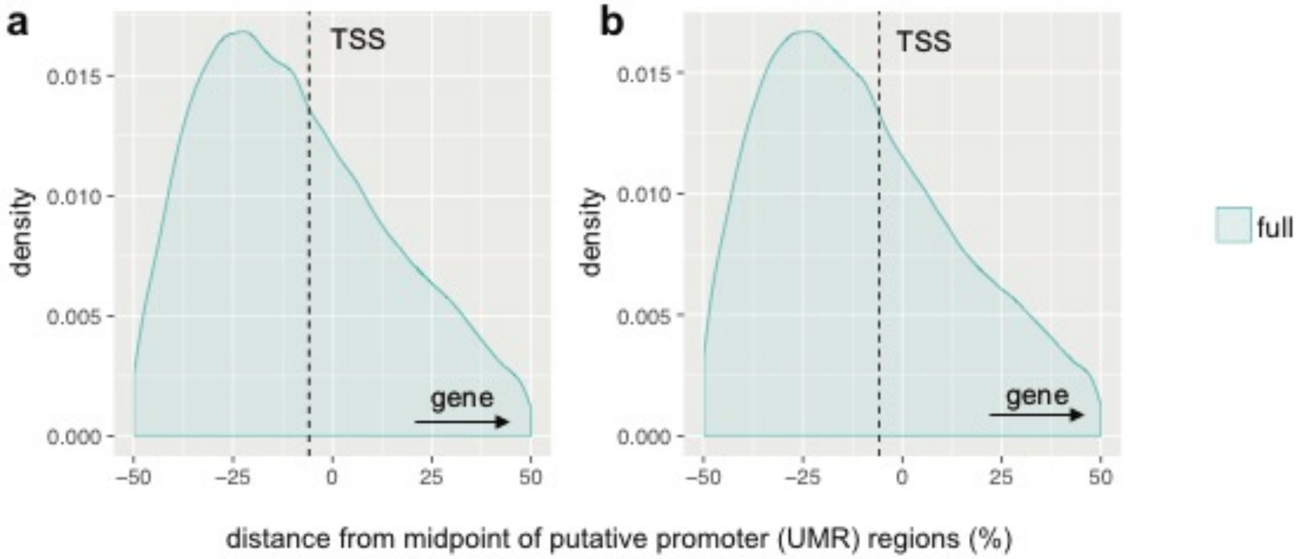

Supplementary Figure 9. Positional mapping of CpGs overlaying Illumina 450K and EPIC array probes within adipose tissue putative promoters. MCC-Seq CpGs overlapping Illumina 450K and EPIC array CpGs and mapping to adipose tissue regulatory promoter regions (UMRs) were further investigated for specific positional trends. Positions of CpGs were tabulated as the percent distance from the midpoint of elements (genomic distance from midpoint (bp)/length of element(bp)*100) and collapsed to summarize positional trends over all assessed elements. Positional trends within UMRs mapping to +/-1.5kb of transcription start sites (TSS) not depicting bivalent gene transcription orientations (taking gene orientation into account) are shown for (a) all CpGs overlaying 450K array CpGs (N=93,648) and (b) all CpGs overlaying EPIC array CpGs (N=137,156).

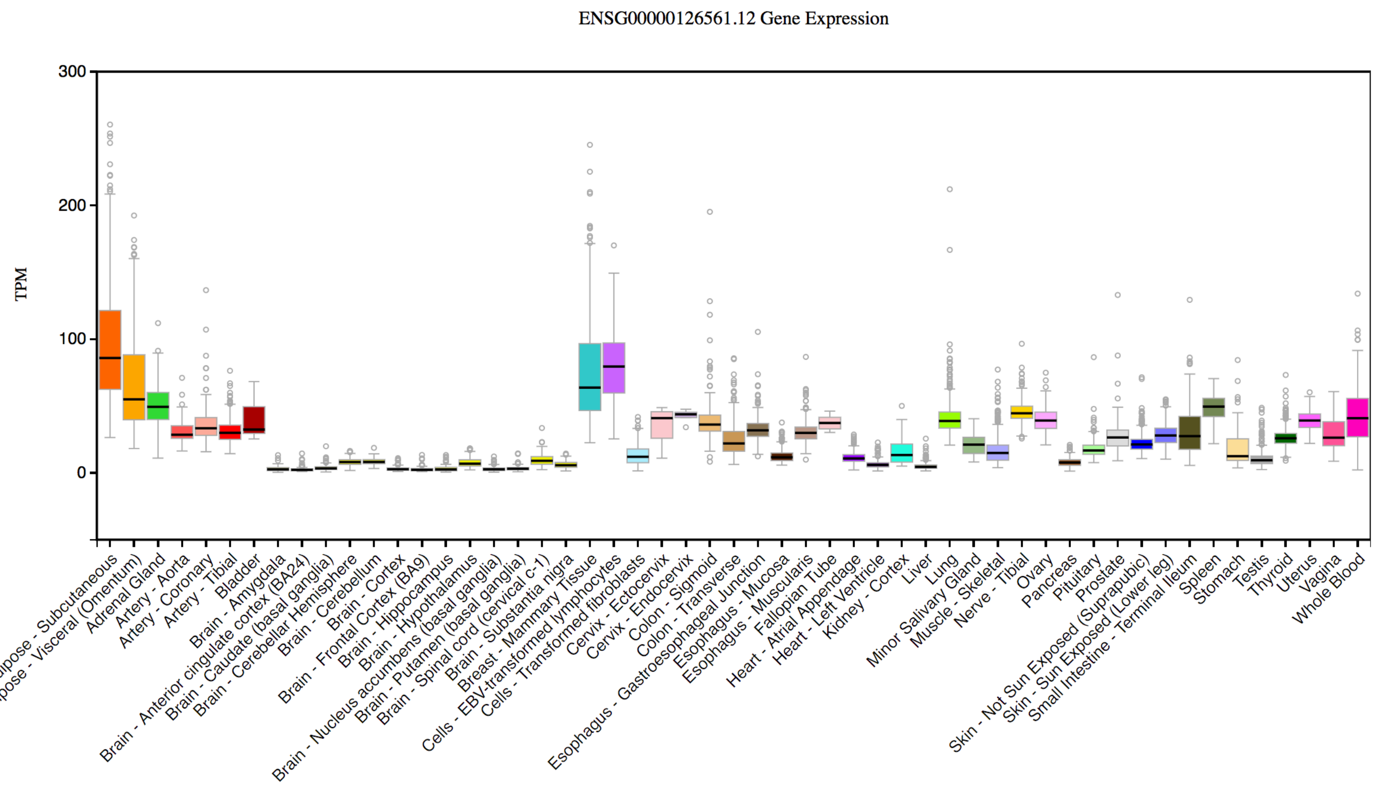

Supplementary Figure 10. Expression profile of *STAT5A* across the multiple tissues in GTEx. Expression levels are assessed in TPM (transcripts per kilobase million; y-axis) for various tissues. Focusing on subcutaneous adipose, visceral adipose and whole-blood tissues, values of 85.865, 54.950 and 40.860 TPM are reported for this gene, respectively (GTEx Portal; October 2018).

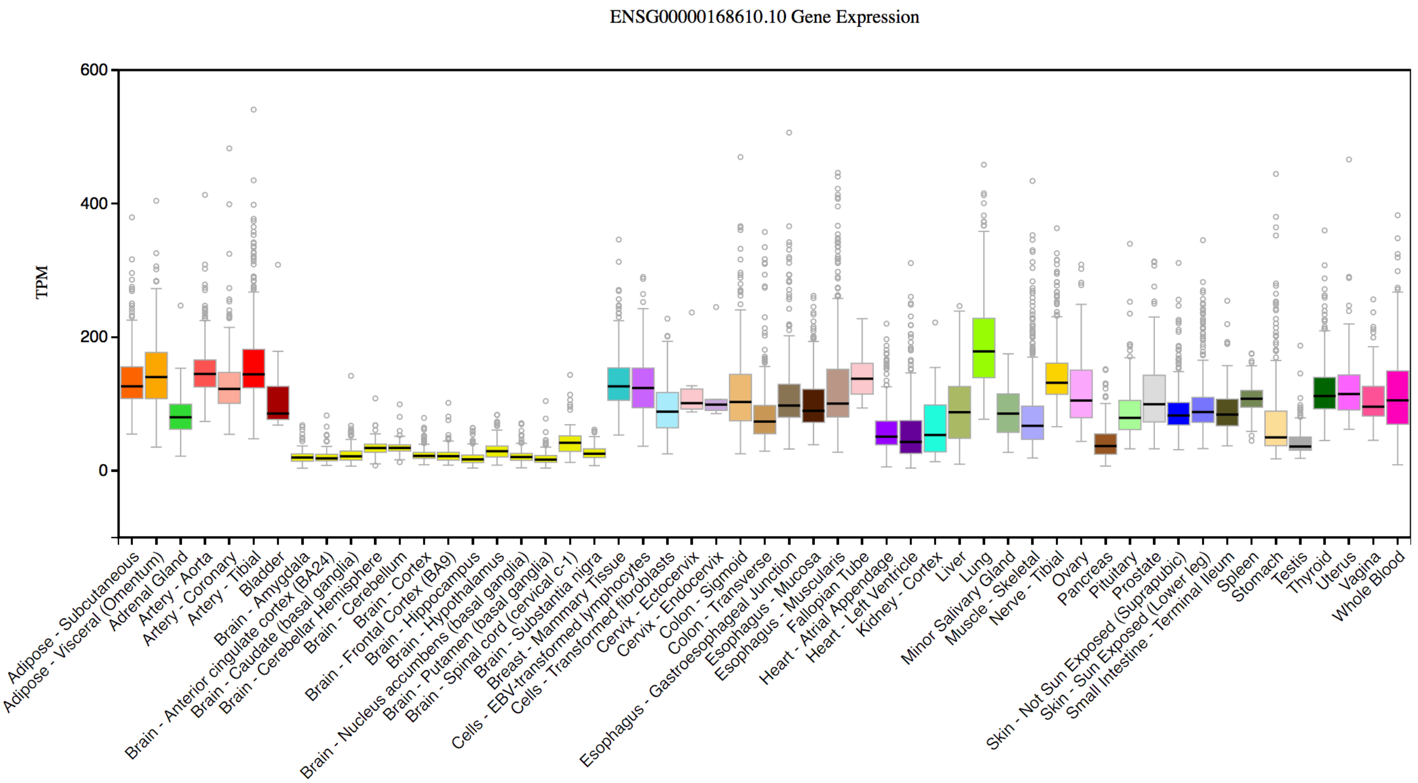

Supplementary Figure 11. Expression profile of *STAT3* across the multiple tissues in GTEx. Expression levels are assessed in TPM (transcripts per kilobase million; y-axis) for various tissues. Focusing on subcutaneous adipose, visceral adipose and whole-blood tissues, values of 126.240, 140.190 and 105.300 TPM are reported for this gene, respectively (GTEx Portal; October 2018).

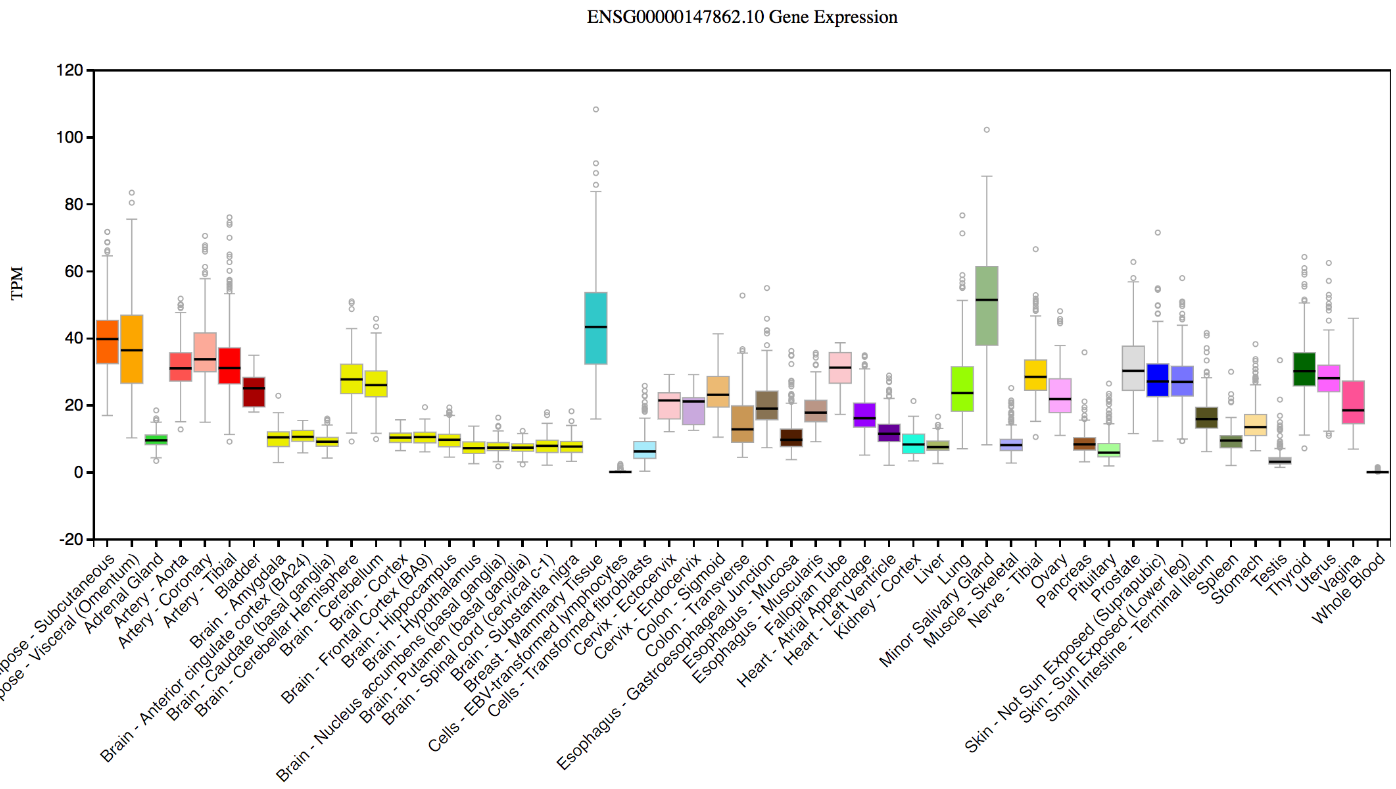

Supplementary Figure 12. Expression profile of *NFIB* across the multiple tissues in GTEx. Expression levels are assessed in TPM (transcripts per kilobase million; y-axis) for various tissues. Focusing on subcutaneous and visceral adipose tissues values of 39.790 and 36.470 TPM are reported for this gene (GTEx Portal; October 2018).

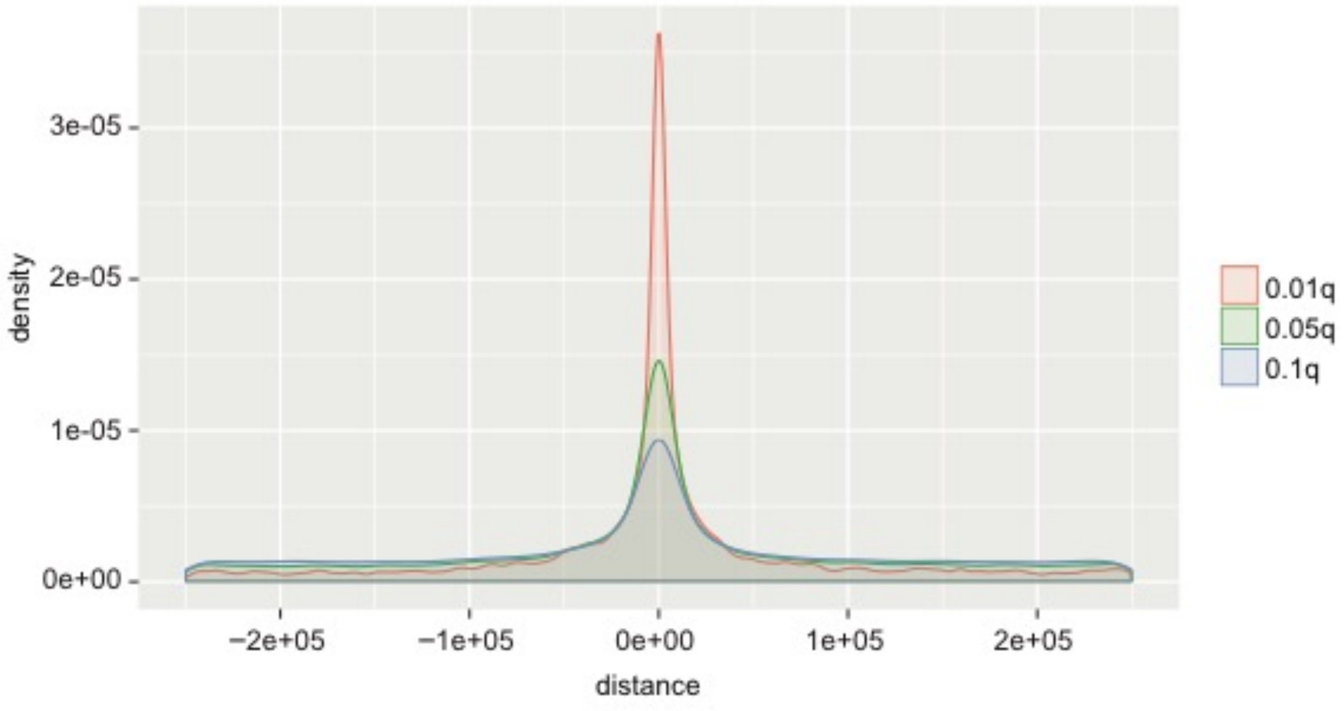

Supplementary Figure 13. Genomic distance between CpGs and their top associated SNP. Adipose tissue SNP-CpG associations (metQTL; +/-250kb) were overlapped with discovery cohort CpGs (N=1,299,494). We depict the genomic distance between the CpGs and their top associated SNP for metQTLs at FDR 10% (N=110,957; $Median_{abs}$=69,303bp), FDR 5% (N=64,240; $Median_{abs}$=46,183bp) and, FDR 1% (N=27,392; $Median_{abs}$=17,230bp) – noting an enrichment of SNPs regulating methylation in the vicinity of their linked CpGs.

Supplementary Tables

Supplementary Table 1. Characteristics of the study cohorts

Trait	Discovery Adipose	MuTHER Adipose	IUCPQ Whole-blood	CARTaGENE Whole-blood
**Study population**	Obese	Normal	Obese	Normal
**N (% female)**	199 (60%)	648 (100%)	206 (55%)	137 (35%)
**Age (years) [SD]**	37.2 [8.8]	58.9 [9.4]	38.9 [9.8]	55.2 [7.8]
**BMI (kg/m^2) [SD]**	53.7 [8.9]	26.7 [4.8]	51.5 [8.8]	26.8 [4.4]
**Triglycerides (mmol/L) [SD]**	1.5 [0.7]	1.1 [0.6]	1.5 [0.6]	1.6 [0.9]
**HDL-C (mmol/L) [SD]**	1.3 [0.3]	1.8 [0.5]	1.3 [0.3]	1.3 [0.4]
**LDL-C (mmol/L) [SD]**	2.8 [0.7]	3.3 [1.0]	2.8 [0.8]	3.0 [0.9]
**Total cholesterol (mmol/L) [SD]**	4.8 [0.8]	5.6 [1.1]	4.8 [0.9]	5.0 [1.0]

Supplementary Table 2. Size and CpG density characterization of adipose regulatory regions

Adipose regulatory region type	Length			Discovery CpG density (CpGs/region)		
	mean (bp)	min (bp)	max (bp)	mean	min	max
LMR	804	100	8688	7	1	29
UMR	2199	233	6992	37	1	355

Supplementary Table 3. Overlap between discovery CpGs versus those on the EPIC and 450K array at adipose regulatory regions

Adipose regulatory region type	Discovery CpGs	EPIC array CpGs		MuTHER array CpGs	
		Total CpGs*	Directly overalpping discovery CpGs*	Total CpGs*	Directly overalpping discovery CpGs*
**LMR**	225,771	38,416 (17%)	23,317 (10%)	13,256 (6%)	9,407 (4%)
**UMR**	696,492	201,113 (29%)	83,159 (12%)	133,755 (19%)	56,422 (8%)

* Percentages are calculated using the total number of discovery CpGs per category as the denominator

Supplementary Table 4. Transcription factor binding site motifs at regions flanking replicated MuTHER lipid-CpGs mapping to UMRs

Rank	Motif Name	Consensus	Entrez	Candidate gene	p-value	Log p-value	q-value (Benjamini)	Target sequences with motifs (%)	Background sequences with motif (%)
1	STAT5(Stat)/mCD4+-Stat5-ChIP-Seq(GSE12346)/Homer	RTTTCTNAGAAA	6776	*STAT5A*	1.00E-04	-1.11E+01	3.90E-03	0.6	0.1
2	STAT1(Stat)/HelaS3-STAT1-ChIP-Seq(GSE12782)/Homer	NATTTCCNGGAAAT	6772	*STAT1*	1.00E-04	-1.02E+01	4.90E-03	0.6	0.1
3	Stat3+il21(Stat)/CD4-Stat3-ChIP-Seq(GSE19198)/Homer	SVYTTCCNGGAARB	6774	*STAT3*	1.00E-03	-8.14E+00	2.58E-02	0.8	0.4
4	NF1(CTF)/LNCAP-NF1-ChIP-Seq(Unpublished)/Homer	CYTGGCABNSTGCCAR	4781	*NFIB*	1.00E-03	-7.40E+00	4.03E-02	0.8	0.3
5	RUNX(Runt)/HPC7-Runx1-ChIP-Seq(GSE22178)/Homer	SAAACCACAG	861	*RUNX1*	1.00E-03	-6.99E+00	4.89E-02	0.7	0.3

Supplementary Table 5. Top canonical pathways for genes modulated by replicated lipid-linked regulatory regions and further linked to the same circulating lipid traits

Ingenuity canonical pathways	P-value	Molecules	Total number of molecules
Gαq Signaling	6.94E-05	*GNA15, CSK, KLB, GNG7*	4
Ephrin B Signaling	1.40E-04	*GNA15, RAC3, GNG7*	3
SAPK/JNK Signaling	4.96E-04	*KLB, RAC3, GNG7*	3
Relaxin Signaling	1.34E-03	*GNA15, KLB, GNG7*	3
Tec Kinase Signaling	1.65E-03	*GNA15, KLB, GNG7*	3

Supplementary Table 6. Top canonical pathways for genes overlapping lipid-linked regulatory regions replicating in whole-blood

Ingenuity canonical pathways	P-value	Molecules	Total number of molecules
Adipogenesis pathway	3.08E-03	*HDAC4, BMP4, AKT1*	3
S-methyl-5-thio-α-D-ribose 1-phosphate Degradation	6.50E-03	*MRI1*	1
Oxidized GTP and dGTP Detoxification	6.50E-03	*NUDT1*	1
Axonal Guidance Signaling	1.71E-02	*BMP4, AKT1, RHOD, GDF7*	4
Ceramide Signaling	1.96E-02	*AKT1, CERK*	2

Supplementary Notes

Supplementary Note 1. List of MuTHER members thanked in the acknowledgements

Kourosh R. Ahmadi, Chrysanthi Ainali, Amy Barrett, Veronique Bataille, Jordana T. Bell, Alfonso Buil, Emmanouil T. Dermitzakis, Antigone S. Dimas, Richard Durbin, Daniel Glass, Neelam Hassanali, Catherine Ingle, David Knowles, Maria Krestyaninova, Cecilia M. Lindgren, Christopher E. Lowe, Eshwar Meduri, Paola di Meglio, Josine L. Min, Stephen B. Montgomery, Frank O. Nestle, Alexandra C. Nica, James Nisbet, Stephen O'Rahilly, Leopold Parts, Simon Potter, Johanna Sandling, Magdalena Sekowska, So-Youn Shin, Kerrin S. Small, Nicole Soranzo, Gabriela Surdulescu, Mary E. Travers, Loukia Tsaprouni, Sophia Tsoka, Alicja Wilk, Tsun-Po Yang & Krina T. Zondervan.
