# Supplementary material for: Dissecting features of epigenetic variants underlying cardiometabolic risk using full-resolution epigenome profiling in regulatory elements
Source: Nat Commun. 2019 Mar 14;10:1209. doi: 10.1038/s41467-019-09184-z (PMC6418220; doi:10.1038/s41467-019-09184-z)
Supplement: Supplementary file 3 — Description of Additional Supplementary Files [file 41467_2019_9184_MOESM3_ESM.pdf]

Description of Additional Supplementary Files

Supplementary Data 1

Description: Associations of TG, HDL, LDL and TC to adipose DNA methylation in the discovery cohort at FDR 10%

Supplementary Data 2

Description: Annotation of replicated lipid-linked adipose regulatory regions

Supplementary Data 3

Description: List of significant association of methylation to gene expression within replicated regulatory regions at FDR 10%

Supplementary Data 4

Description: Replicated lipid-linked adipose regulatory regions with three-way associations validation at FDR 10%

Supplementary Data 5

Description: Dissection of adipose lipid-linked regulatory regions shared across to whole-blood

Supplementary Data 6

Description: Replication of tissue-shared lipid-linked regulatory regions in an independent population-based whole-blood cohort

Supplementary Data 7

Description: Co-regulation network between an adipose-unique TG-linked enhancer region, *GNA15* and *GNG7*

Supplementary Data 8

Description: Genetic regulation at an adipose-specific and HDL-linked enhancer region mapping to an intron of *GALNT2*
